# Supplementary figures and images for: Diverse RNA interference strategies in early-branching metazoans
Source: BMC Evol Biol. 2018 Nov 1;18:160. doi: 10.1186/s12862-018-1274-2 (PMC6211395; doi:10.1186/s12862-018-1274-2)

Additional file 1

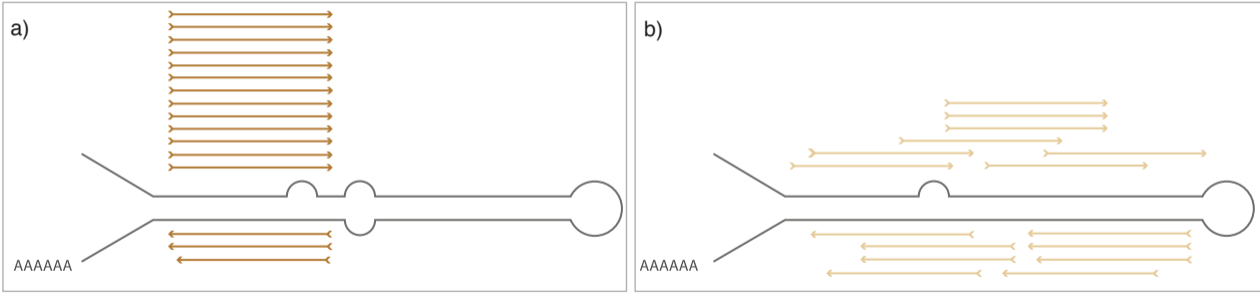

Supplement: Supplementary file 1 — Demonstration of High Uniformity and Low Uniformity sRNA clusters. Two hypothetical hairpin RNAs demonstrating the difference between a high uniformity and a low uniformity clustering. In (a), a total of 16 reads composed of just three distinct reads map to a hairpin RNA giving a uniformity index of 5.3. In (b), 16 reads also map to a hairpin RNA but these are composed of 12 distinct reads resulting in a uniformity index of just 1.3. The high uniformity cluster (a) is composed of an equal number of reads to the low uniformity cluster (b) however these reads are less evenly distributed along the length of the source hairpin RNA. (PDF 304 kb) [file 12862_2018_1274_MOESM1_ESM.pdf]

a)

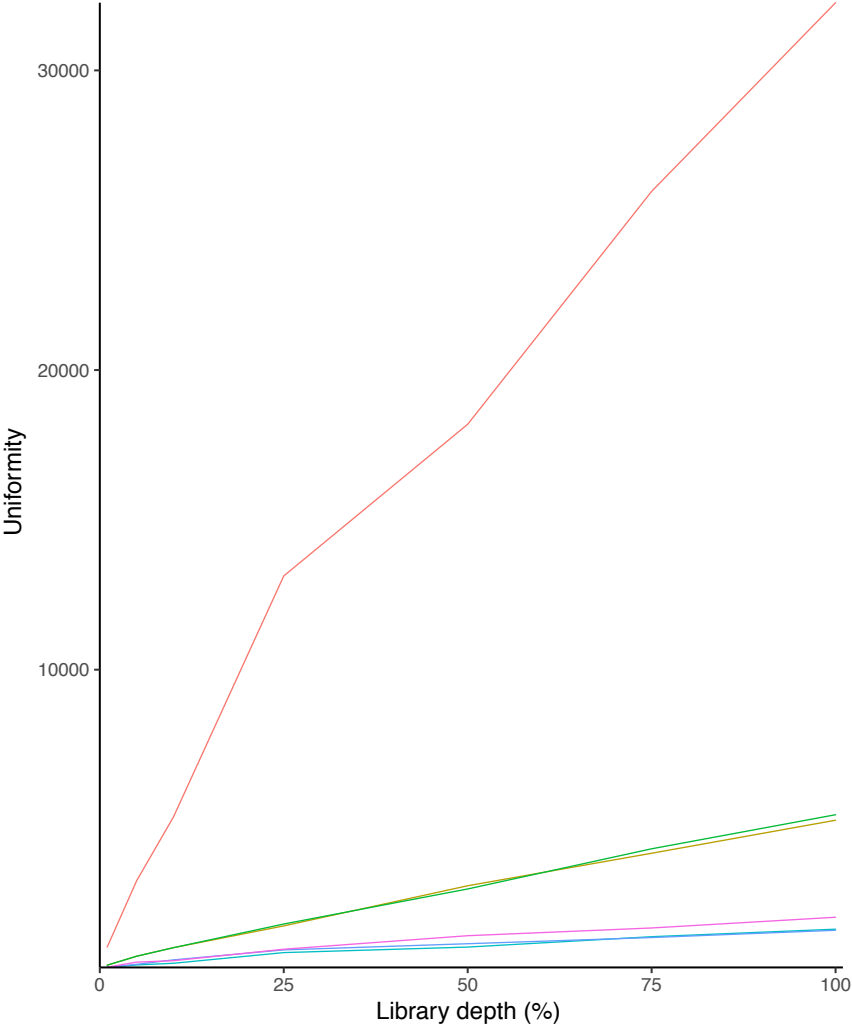

b)

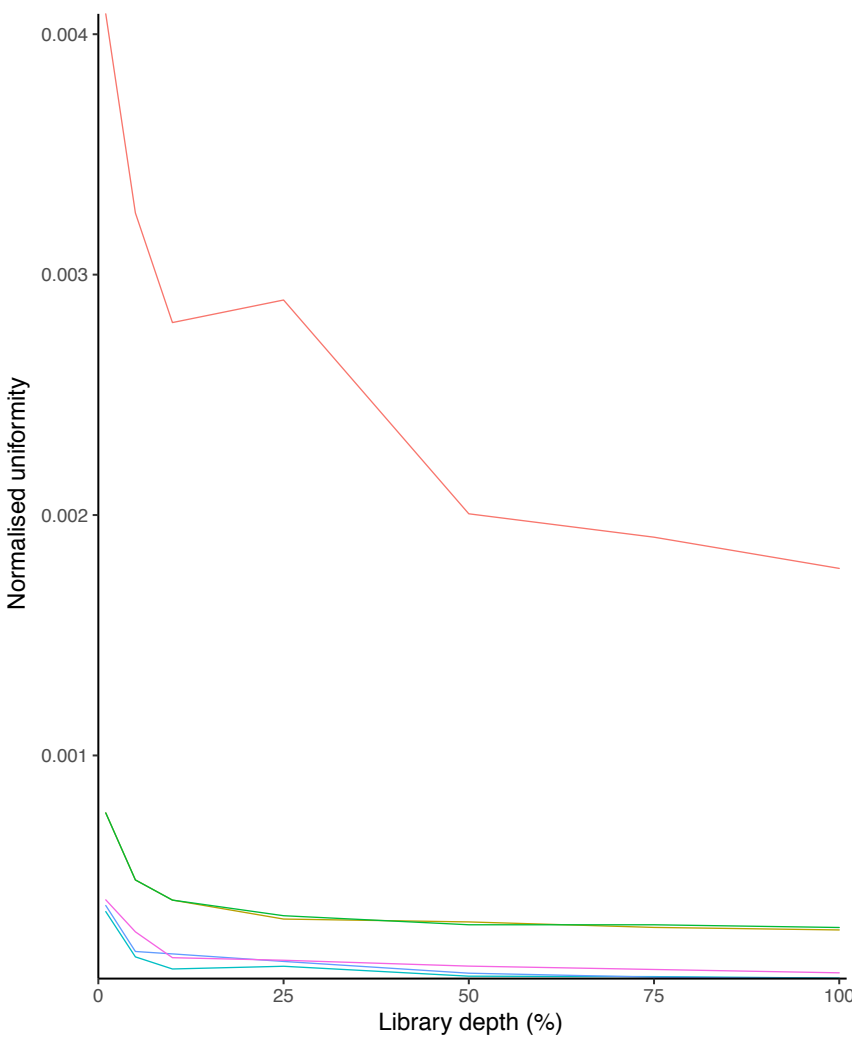

Supplement: Supplementary file 2 — Effect of library depth on uniformity index. Random sampling of reads from the Amphimedon juvenile library (1, 5, 10, 25, 50, 75, 100%) show a trend towards increasing UI for high uniformity miRNA clusters as library depth increases (a). Dividing the UI by the library depth acts to normalise these values (b). Library depth normalised UIs can be more accurately compared between libraries. (PDF 138 kb) [file 12862_2018_1274_MOESM2_ESM.pdf]

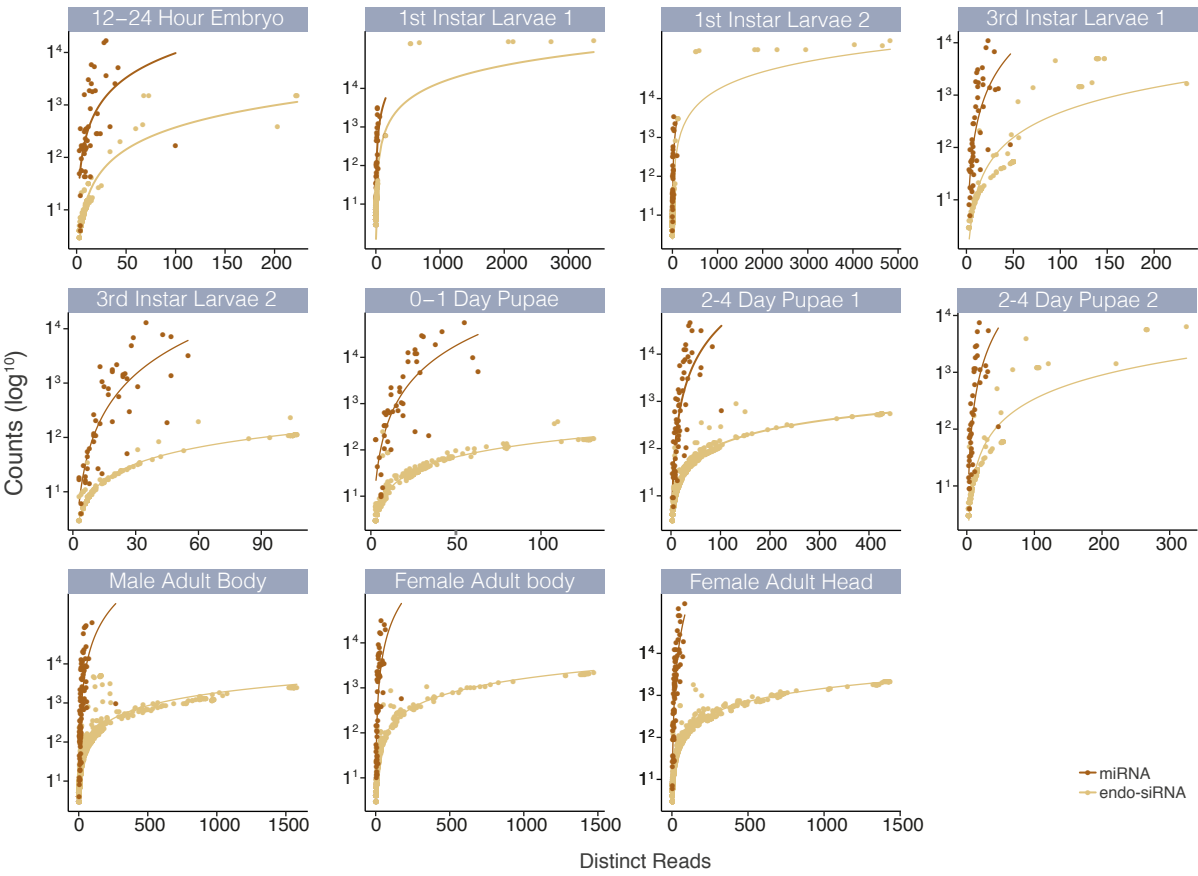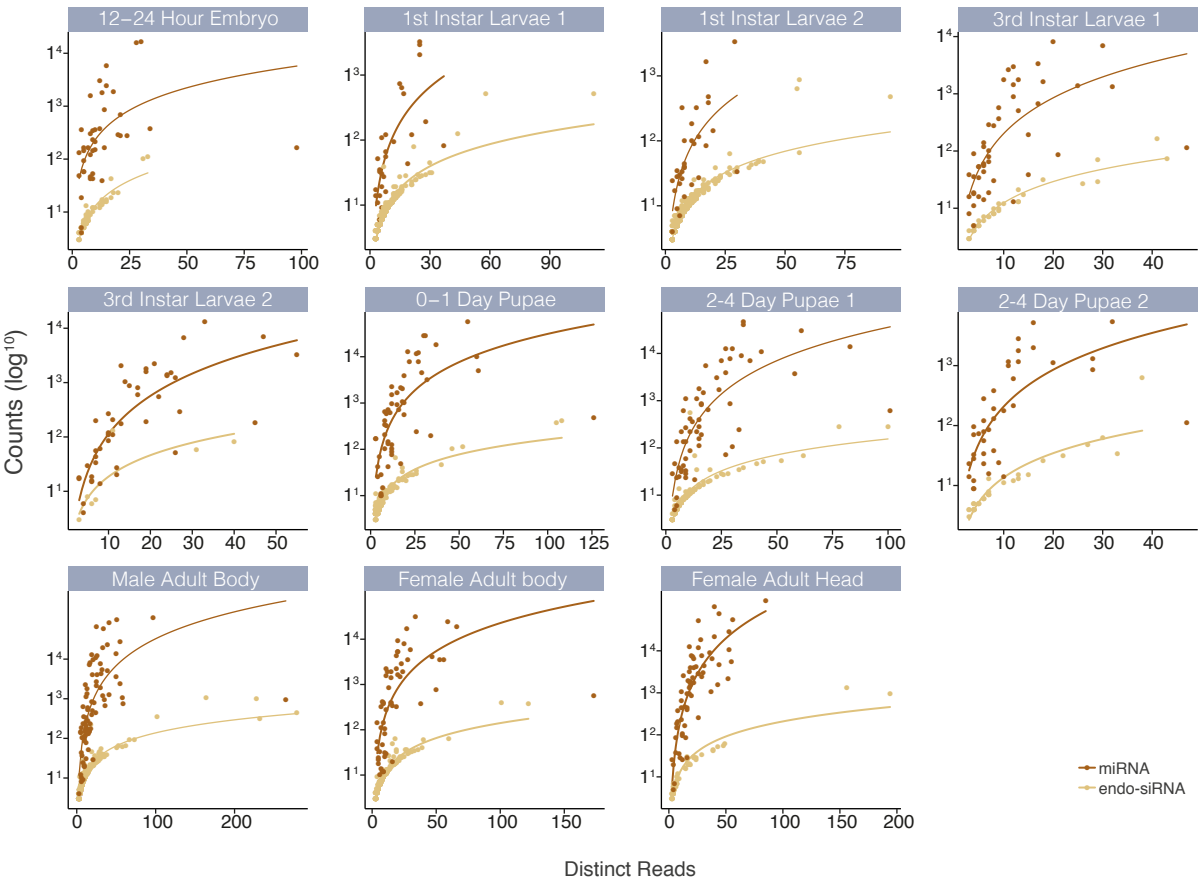

Supplement: Supplementary file 3 — Uniformity of Drosophila endo-siRNA and miRNA clusters. Endo-siRNA clusters (yellow) display a consistently lower uniformity of small RNA expression (ratio of total read counts:distinct reads) in comparison to miRNA clusters (red) for both unique clusters (above) and multi-mapping clusters (below). (PDF 3341 kb) [file 12862_2018_1274_MOESM3_ESM.pdf]

Additional file 6

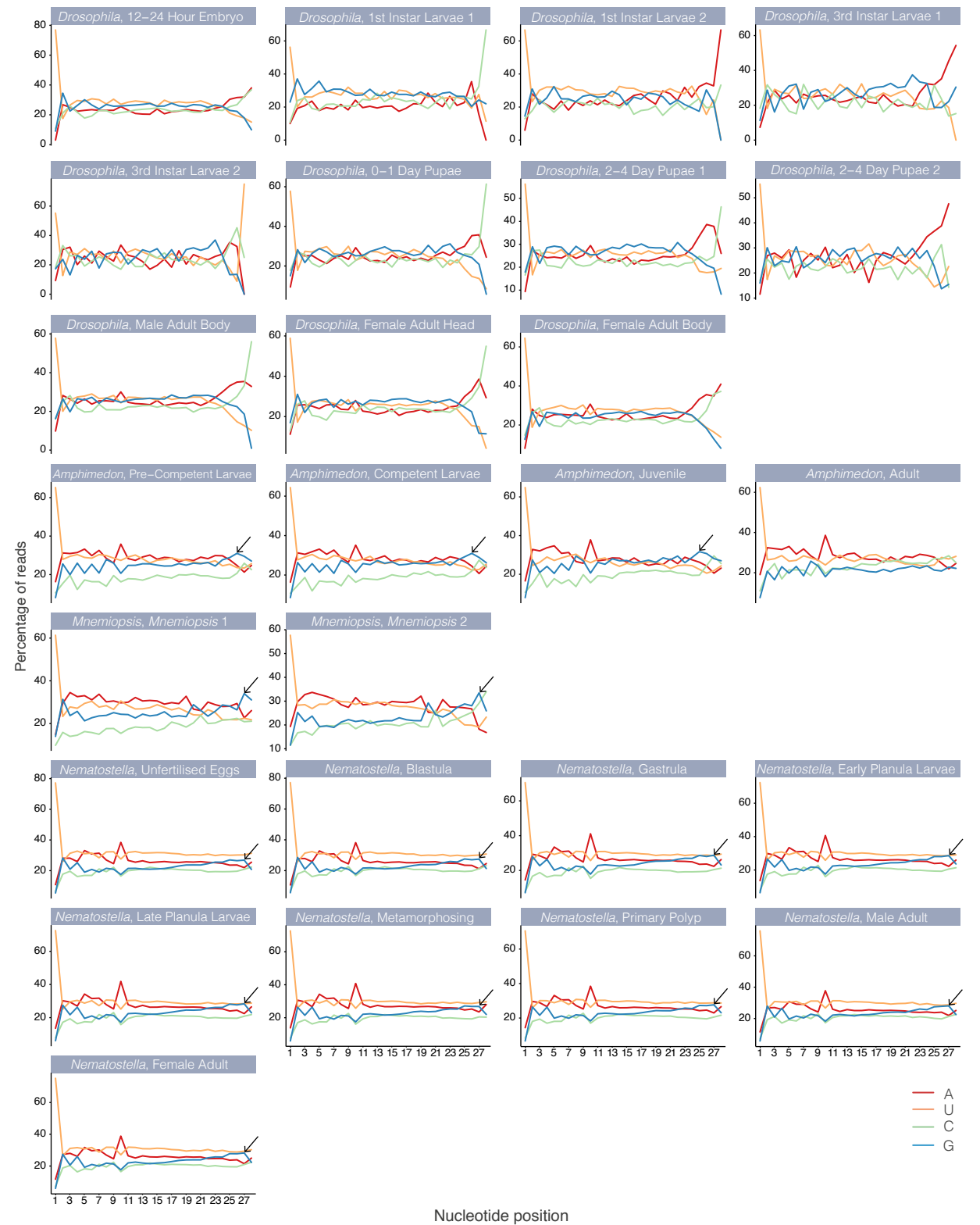

Supplement: Supplementary file 6 — Nucleotide biases of piRNA clusters. Nucleotide biases along the length of all sRNAs mapping to predicted piRNA clusters. sRNAs were anchored at their 5′ nucleotide and biases are displayed as a percentage the contribution of each nucleotide at each position. Of note is the tendency for a uracil at position 1 and an adenosine at position 10 in most libraries that together comprise the ping-pong piRNA biogenesis signature. Arrows indicate guanosine enrichments downstream of position 25. (PDF 323 kb) [file 12862_2018_1274_MOESM6_ESM.pdf]

Additional file 7

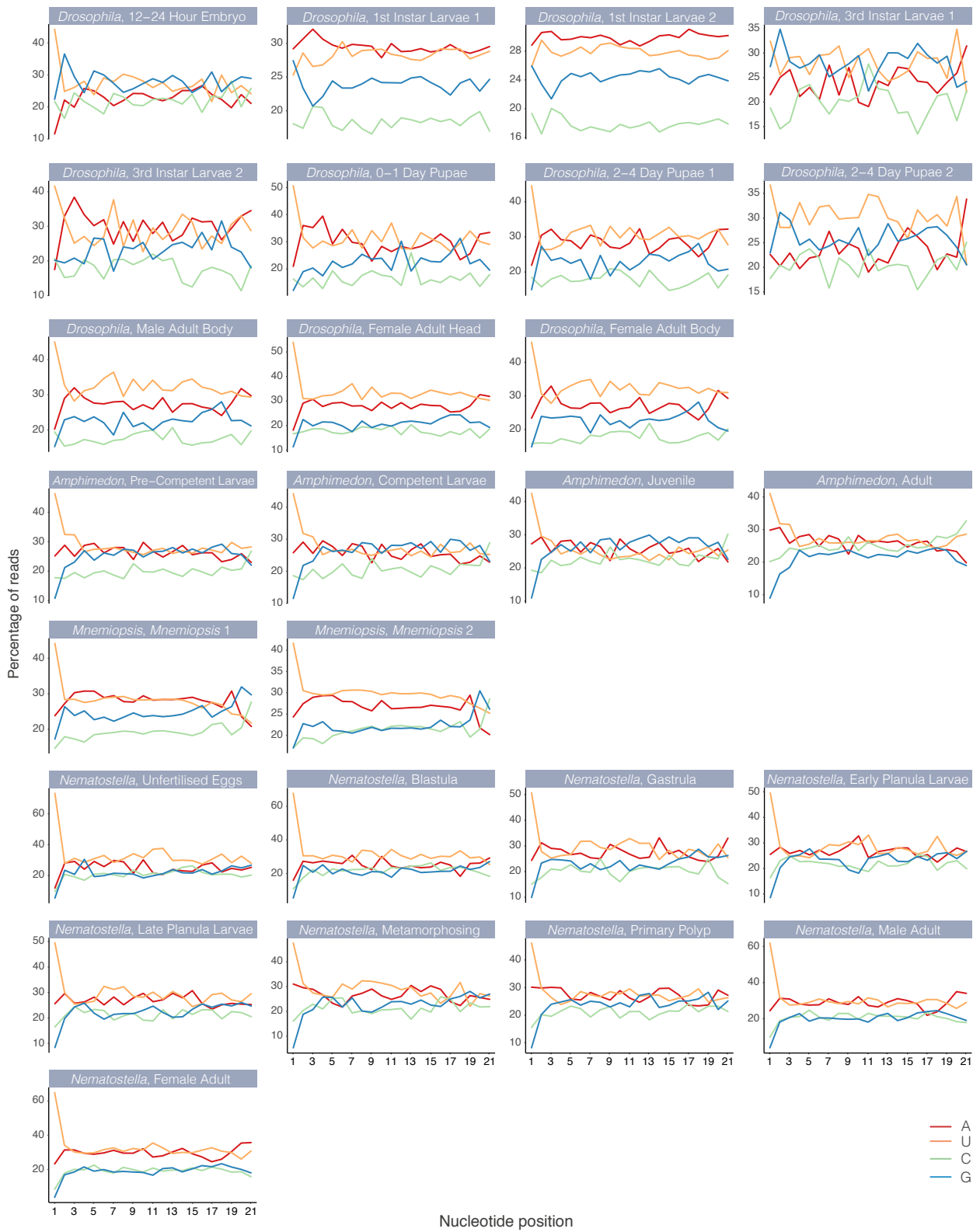

Supplement: Supplementary file 7 — Nucleotide biases of endo-siRNA clusters. Nucleotide biases along the length of all sRNAs mapping to predicted endo-siRNA clusters. sRNAs were anchored at their 5′ nucleotide and biases are displayed as a percentage of the contribution of each nucleotide at each position. Of note is the tendency for a uracil at position 1 which is present in all libraries except the Drosophila 1st instar larval libraries. (PDF 328 kb) [file 12862_2018_1274_MOESM7_ESM.pdf]

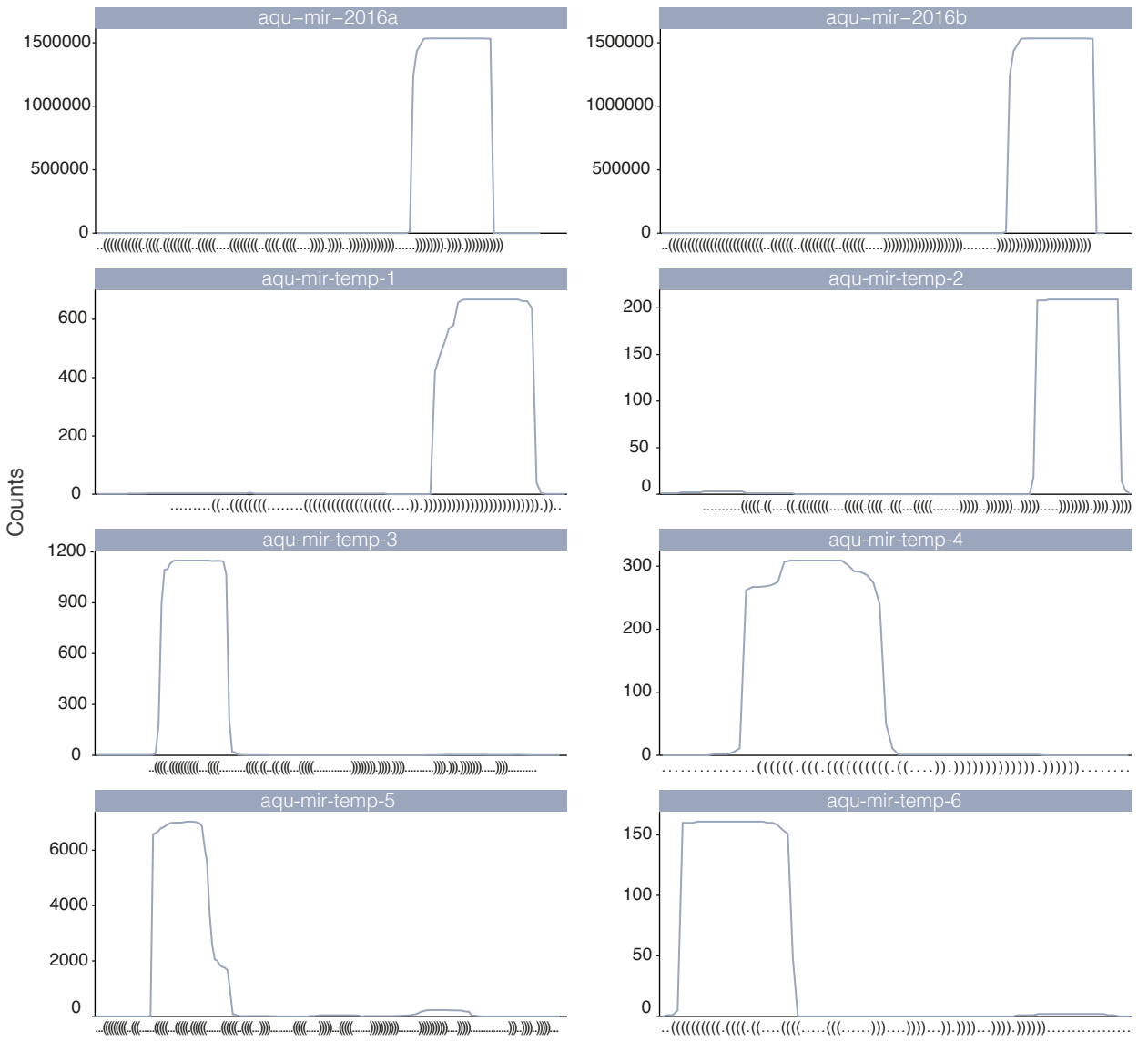

Supplement: Supplementary file 8 — New Amphimedon miRNA candidates. Wiggle plots and predicted secondary structures of mapped reads across the length of previously described miRNA miR-2016a, the newly identified miR-2016b and six novel miRNA candidates (aqu-mir-temp-1-6). For each cluster, the library with the most mapped reads to each loci was used to construct the graph. (PDF 483 kb) [file 12862_2018_1274_MOESM8_ESM.pdf]

Additional file 9

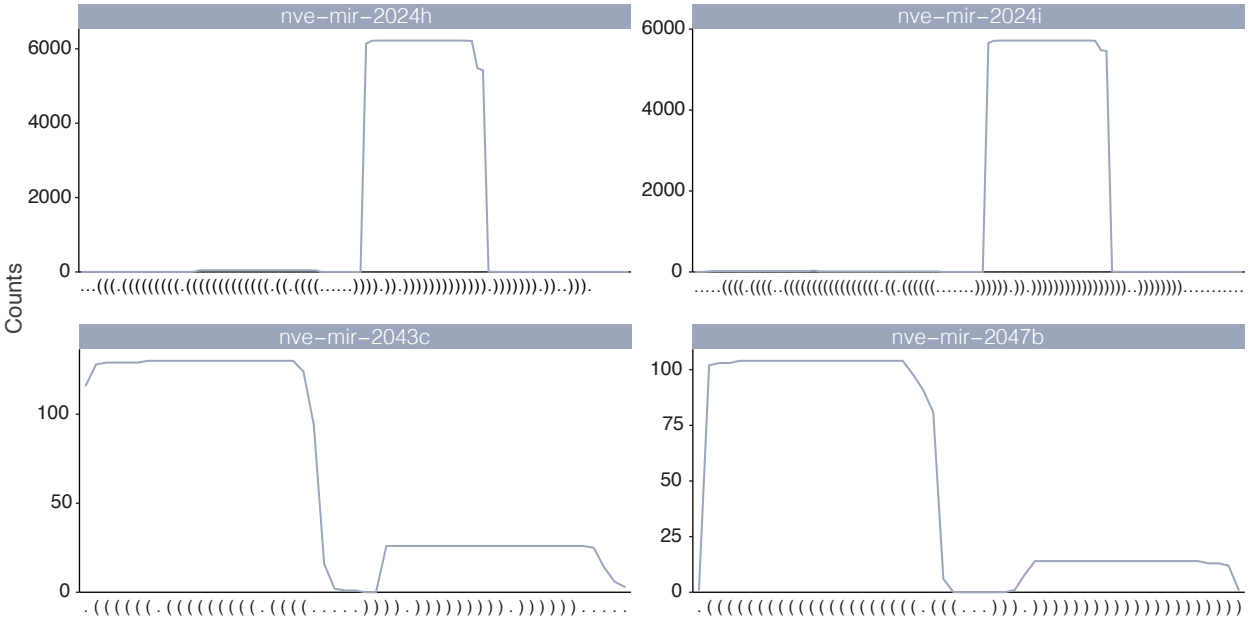

Supplement: Supplementary file 9 — New Nematostella miRNA candidates. Wiggle plots and predicted secondary structures of four newly identified miRNAs in the sea anemone. All four miRNAs are new copies of previously identified miRNAs. (PDF 205 kb) [file 12862_2018_1274_MOESM9_ESM.pdf]

Additional file 12

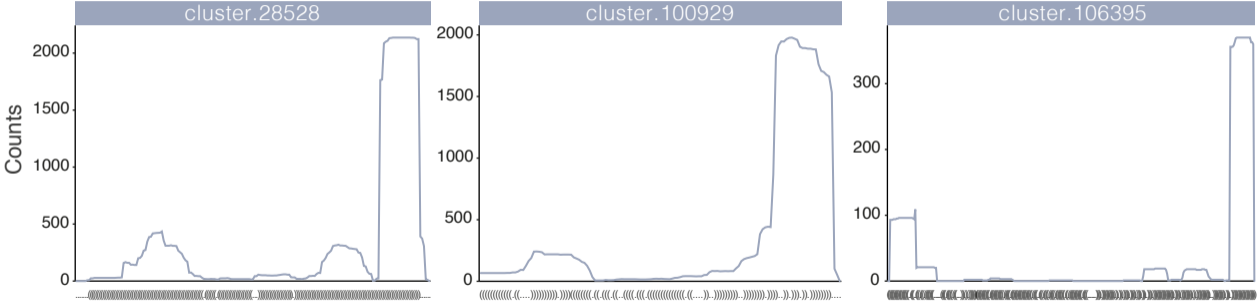

Supplement: Supplementary file 12 — Amphimedon endogenous hairpin RNAs. Wiggle plots and predicted secondary structure of three long highly complementary endo-siRNAs from Amphimedon with unevenly distributed mapped sRNA populations. (PDF 348 kb) [file 12862_2018_1274_MOESM12_ESM.pdf]

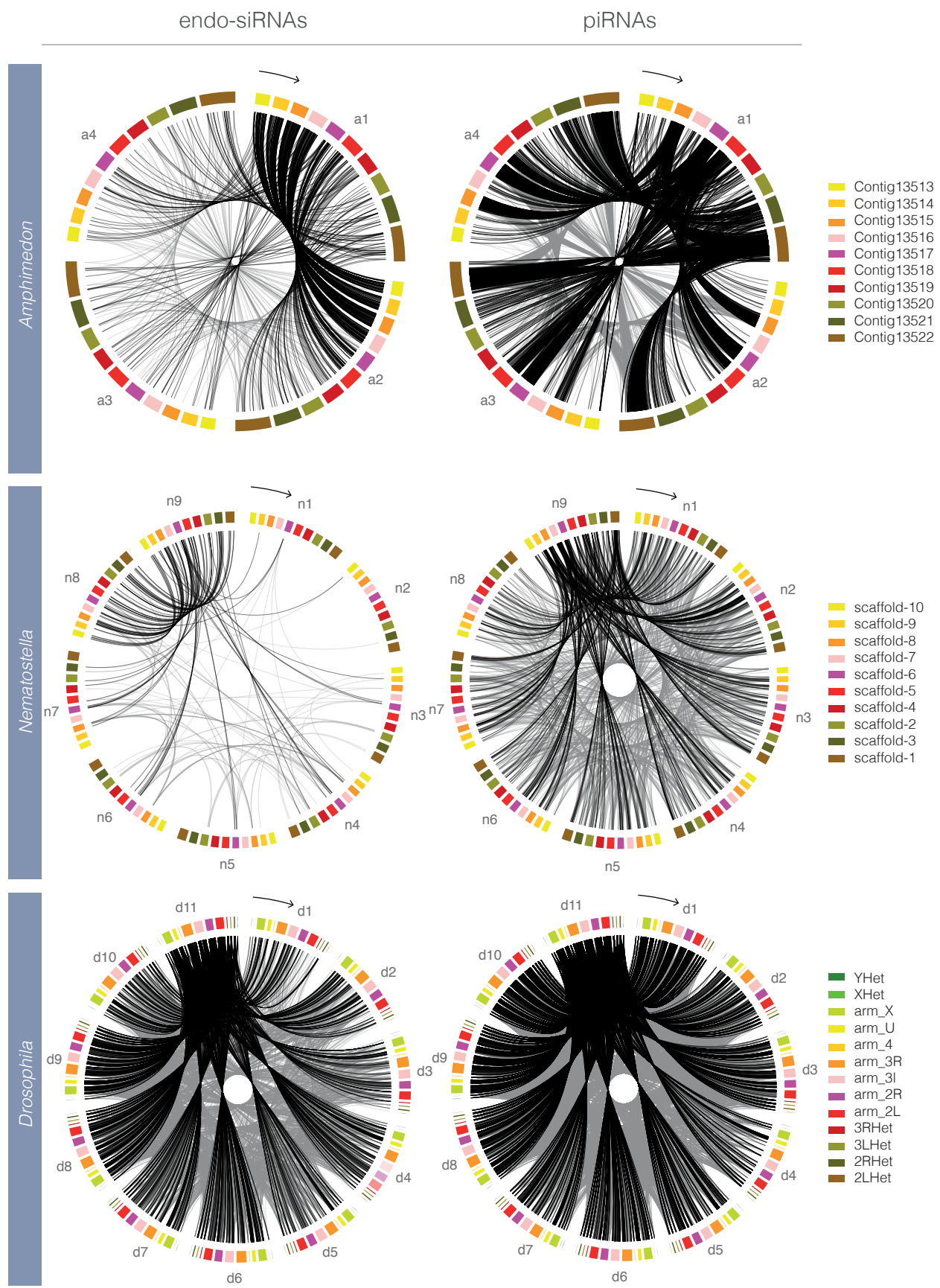

Supplement: Supplementary file 13 — Co-expression of multi-mapping endo-siRNA and piRNA clusters across development. Each plot is divided into groups of coloured scaffolds/chromosomes, each of which represents a developmental stage. For each plot, the earliest developmental stage is marked with an arrow indicating the chronological order of the following developmental stages. Links between scaffolds/chromosomes indicate co-expression from a particular endo-siRNA or piRNA cluster in the two linked developmental stages. For Drosophila, all chromosomes are represented while for Amphimedon and Nematostella, the ten largest genomic scaffolds were used. Beginning with the developmental stage indicated by the arrow, the stages for Amphimedon, Nematostella and Drosophila are as per Fig. 1. For each species, the links shared with a single developmental stage are coloured black for emphasis while the rest are coloured grey. For Amphimedon the emphasised stage is the pre-competent larvae, for Nematostella the female adult and for Drosophila, the female adult head. (PDF 23669 kb) [file 12862_2018_1274_MOESM13_ESM.pdf]
