# Supplementary material for: Diverse RNA interference strategies in early-branching metazoans
Source: BMC Evol Biol. 2018 Nov 1;18:160. doi: 10.1186/s12862-018-1274-2 (PMC6211395; doi:10.1186/s12862-018-1274-2)
Supplement: Supplementary file 5 — Supplementary Methods. Detailed methods (DOCX 66 kb) [file 12862_2018_1274_MOESM5_ESM.docx]

**SUPPLEMENTARY METHODS**

**Biological sampling of *Amphimedon queenslandica***

RNA was obtained from larval, juvenile and adult *Amphimedon queenslandica* inhabiting Shark Bay, Heron Island Reef on the southern Great Barrier Reef, Australia (Latitude − 23.44, Longitude 151.92) as previously described [1]. Pre-competent larvae and competent [2,3] were sampled 0-2 and 8-10 hours post-emergence (0-2 and 8-10 hpe), respectively. Juveniles were samples about 3 days after induction of settlement with the introduction of a coralline algae settlement cue [3]. RNA was extracted from pools of between 30 and 50 pre-competent larvae, competent larvae and juveniles.

**Analysis of endo-siRNA cluster overlap with reference database**

To test whether the identified endo-siRNA loci overlapped with previously reported endo-siRNA loci more often than would be expected by chance, the locations of the endo-siRNA clusters were randomised 100,000 times (randomBed, BEDTools v2.25.0) [4] and then intersected with the reference dataset using overlapSelect [5]. This showed a distribution of overlapping clusters with a mean of 119.5, a standard deviation of 11.6 and maximum and minimum values of 173 and 75 (Fig. 1).


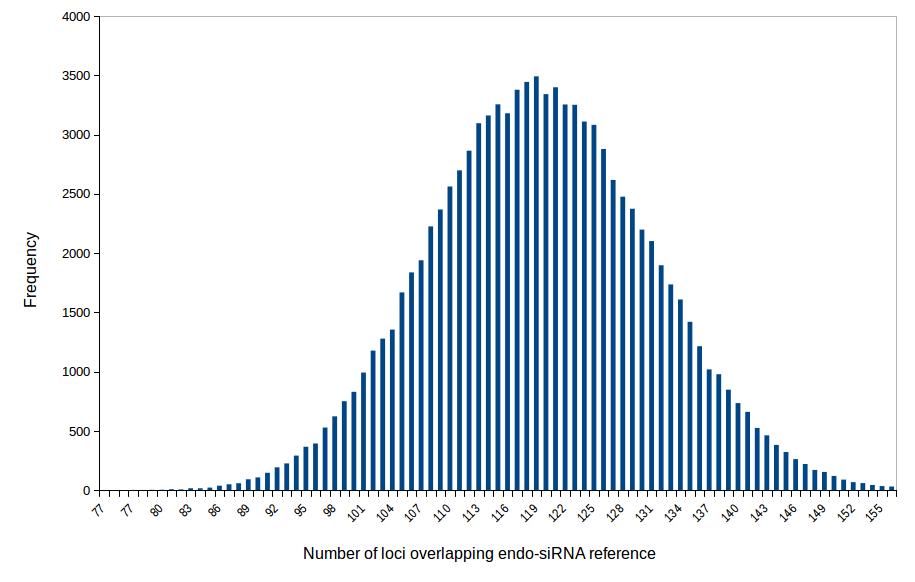


**Fig. 1 Frequency distribution of intersections of endo-siRNA reference database with randomised endo-siRNA loci.**

**REFERENCES**

1. Leys SP, Larroux C, Gauthier M, Adamska M, Fahey B, Richards GS, Degnan SM, Degnan BM. 2008. Isolation of *Amphimedon* developmental material. Cold Spring Harbor Protocols 2008:pdb prot5095. doi: 10.1101/pdb.prot5095

2. Jackson D, Leys SP, Hinman VF, Woods R, Lavin MF, Degnan BM. Ecological regulation of development: induction of marine invertebrate metamorphosis. Int. J. Dev. Biol. 2002;46:679–86.

3. Degnan SM, Degnan BM. The initiation of metamorphosis as an ancient polyphenic trait and its role in metazoan life-cycle evolution. Philos. Trans. R. Soc**.** Lond. B. Biol. Sci. 2010;365:641–51.

4. Quinlan AR, Hall IM. BEDTools: a flexible suite of utilities for comparing genomic features. Bioinformatics. 2010;26:841–2.

5. Kuhn RM, Haussler D, Kent WJ. The UCSC genome browser and associated tools. Brief. Bioinform. 2013;14:144–61.
