# Supplementary material for: Diverse RNA interference strategies in early-branching metazoans
Source: BMC Evol Biol. 2018 Nov 1;18:160. doi: 10.1186/s12862-018-1274-2 (PMC6211395; doi:10.1186/s12862-018-1274-2)
Supplement: Supplementary file 11 — miRDeep2 identification of new Nematostella miRNA candidates. Results of miRDeep2 annotation of the newly identified miRNA candidates from Nematostella. (a) nve-miR-temp-1, (b) nve-miR-temp-2, (c) nve-miR-temp-3, (d) nve-miR-temp-4. (PDF 2157 kb) [file 12862_2018_1274_MOESM11_ESM.pdf]

Additional file 11

(a)

Provisional ID	: scaffold_364_28565
Score total	: 2.7
Score for star read(s)	: -1.3
Score for read counts	: 0
Score for mfe	: 2.4
Score for randfold	: 1.6
Score for cons. seed	:
Total read count	: 24
Mature read count	: 23
Loop read count	: 0
Star read count	: 1

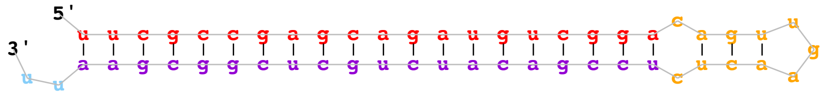

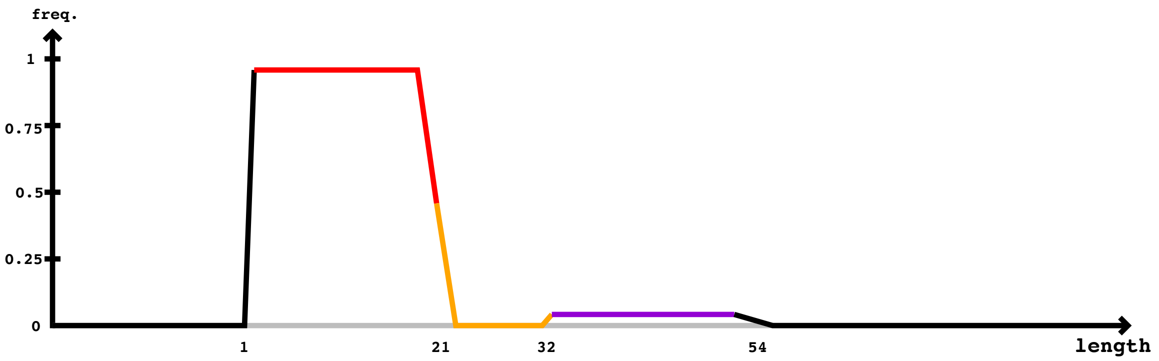


```
                     Mature                         Star
5'- gucaaaauaacugcuuaaauucgccgagcagaugucggacaguugaacucuccgacaucugcucggcgaauugagcaguuacgaauuuacucccggauuuucaaaacgca  -3'   obs
    gucaaaauaacugcuuaaauucgccgagcagaugucggacaguugaacucuccgacaucugcucggcgaauugagcaguuacgaauuuacucccggauuuucaaaacgca         exp
    .......(((((((((((.((((((((((((((((((((.(((...))).))))))))))))))))))))))))))))))).((((((......))))))..........    reads   mm   sample
    ...................uucgccUagcagaugucg.........................................................................    1       1    AdM
    ...................uucgccgagcagaugucg.........................................................................    5       0    AdM
    ...................uucgccgagcagaugucgg........................................................................    5       0    AdM
    ...................uucgccgagcagauUucgg........................................................................    1       1    AdM
    ...................uucgccgagcagaugucgga.......................................................................    11      0    AdM
    ..................................................uccgacaucugcucggcgaa........................................    1       0    AdM
```

Additional file 11

(b)

Provisional ID	:	scaffold_14_2300
Score total	:	68.2
Score for star read(s)	:	3.9
Score for read counts	:	60.8
Score for mfe	:	1.9
Score for randfold	:	1.6
Score for cons. seed	:	
Total read count	:	131
Mature read count	:	108
Loop read count	:	0
Star read count	:	23

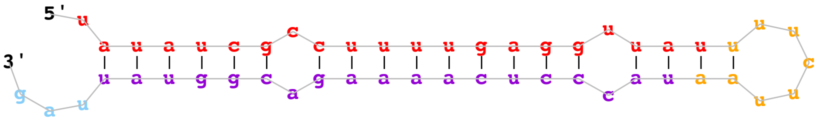

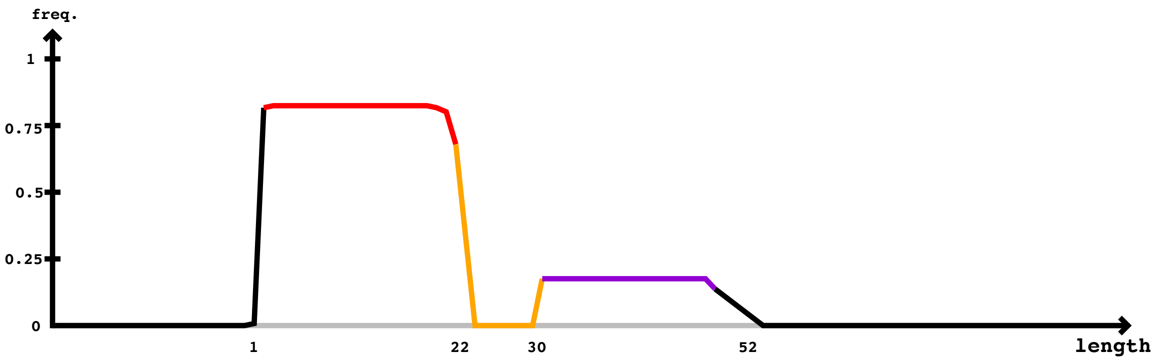

Mature **Star**

```
5'- guaucuaauaucgaaucucuuauaucgccuuuugagguuauuuucuuaauacccucaaaagacgguauuagagauuacacagcuaaacaaucgcugugugaagaugcagu   -3'   obs
    guaucuaauaucgaaucucuuauaucgccuuuugagguuauuuucuuaauacccucaaaagacgguauuagagauuacacagcuaaacaaucgcugugugaagaugcagu         exp
    guaucuaauaucgaaucucuuauaucgccuuuugagguuauuuucuuaauacccucaaaagacgguauuagagauuacacagcuaaacaaucgcugugugaagaugcagu         known
    ((((((.......(((((((.((((((.(((((((((.((((.....)))).))))))))).)))))).)))))))(((((((.........)))))))..))))))...   reads   mm   sample

    ...................Guauaucgccuuuugagguuau.....................................................................   1   1   AdM
    ....................uauaucgccuuuugaggu........................................................................   1   0   AdM
    ....................uauaucgccuuuugagguu.......................................................................   2   0   AdM
    ....................uauaucgccuuuugagguua......................................................................   15  0   AdM
    ....................uauaucgccuuuugagCuua......................................................................   1   1   AdM
    ....................uauaucgccuuuugaggCuau.....................................................................   1   1   AdM
    ....................uauaucgccuuuuCagguuau.....................................................................   1   1   AdM
    ....................uauaucgAcuuuugagguuau.....................................................................   1   1   AdM
    ....................uauaucgccuuuugagguuau.....................................................................   70  0   AdM
    ....................uauaucgccuuuuAagguuau.....................................................................   2   1   AdM
    ....................uauaucgAcuuuugagguuauu....................................................................   1   1   AdM
    ....................uauaucgccuuuugagguuauu....................................................................   10  0   AdM
    ....................uaGaucgccuuuugagguuauu....................................................................   1   1   AdM
    .....................auaAcgccuuuugagguuau.....................................................................   1   1   AdM
    .................................................uacccucaaaagacggua...........................................   5   0   AdM
    .................................................uacccucaaaagacgguau..........................................   8   0   AdM
    .................................................uacccucaaaagacgguauC.........................................   1   1   AdM
    .................................................uacccucaaaagacgguauu.........................................   1   0   AdM
    .................................................uaccAucaaaagacgguauu.........................................   1   1   AdM
    .................................................uacccucaaaagacgguauua........................................   2   0   AdM
    .................................................uaAccucaaaagacgguauua........................................   1   1   AdM
    .................................................uacccucaaaagacgguauuag.......................................   2   0   AdM
    .................................................uacccucaaaagacgguauuaga......................................   1   0   AdM
    .................................................uacccucaaaagacgguauuagU......................................   1   1   AdM
```

Additional file 11

(c)

Provisional ID	:	scaffold_1056_34657
Score total	:	3529.4
Score for star read(s)	:	3.9
Score for read counts	:	3522
Score for mfe	:	1.9
Score for randfold	:	1.6
Score for cons. seed	:	
Total read count	:	6920
Mature read count	:	6899
Loop read count	:	0
Star read count	:	21

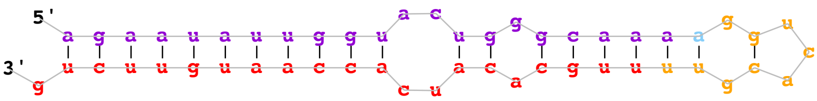

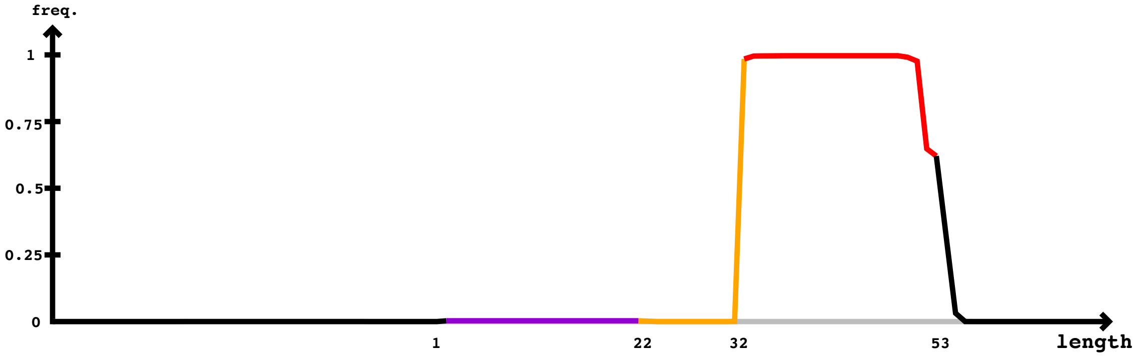

Star Mature

```
5'- auuaugaggaggauuauaaagaucagguagacuugcuucagaauauugguacugggcaaaaggucacguuuugcacaucaccaauguucugaggcacuucugcugaag  -3'  obs
    auuaugaggaggauuauaaagaucagguagacuugcuucagaauauugguacugggcaaaaggucacguuuugcacaucaccaauguucugaggcacuucugcugaag       exp
    auuaugaggaggauuauaaagaucagguagacuugcuucagaauauugguacugggcaaaaggucacguuuugcacaucaccaauguucugaggcacuucugcugaag       known
    .((((((......))))))...((((.((((..(((((((((((((((((..((.((((((.(...).)))))).))..)))))))))))))))))..))))))))..  reads  mm  sample

    ............auuauaaagaucagguaga.............................................................................  1   0  AdM
    .......................................agaauauugguacugggcaa.................................................  1   0  AdM
    .......................................agaauauugguacugUgcaaa................................................  1   1  AdM
    .......................................agaauauugguacugggcGaa................................................  1   1  AdM
    .......................................agaauauugguacugggcaaa................................................  16  0  AdM
    .......................................agaauauugguacuggUcaaa................................................  1   1  AdM
    ........................................gaauauugguacugggcaaa................................................  1   0  AdM
    .....................................................................Auugcacaucaccaauguuc...................  2   1  AdM
    .....................................................................Cuugcacaucaccaauguuc...................  1   1  AdM
    ......................................................................uuUcacaucaccaaugu.....................  1   1  AdM
    ......................................................................uugcacaucaccaaugG.....................  1   1  AdM
    ......................................................................uugcacaucaccaaugu.....................  33  0  AdM
    ......................................................................uugcacauAaccaaugu.....................  1   1  AdM
    ......................................................................Cugcacaucaccaaugu.....................  1   1  AdM
    ......................................................................uugcacaucacAaaugu.....................  1   1  AdM
    ......................................................................uugcacaucaccaauguA....................  1   1  AdM
    ......................................................................uugcacauAaccaauguu....................  1   1  AdM
    ......................................................................uugcacaucaccaauguu....................  92  0  AdM
    ......................................................................uGgcacaucaccaauguu....................  1   1  AdM
    ......................................................................uugcacaGcaccaauguu....................  1   1  AdM
    ......................................................................uugUacaucaccaauguu....................  1   1  AdM
    ......................................................................uugcacaucaccaaugAu....................  1   1  AdM
    ......................................................................uuUcacaucaccaauguu....................  1   1  AdM
    ......................................................................uugcacaucacUaauguu....................  1   1  AdM
    ......................................................................Cugcacaucaccaauguu....................  1   1  AdM
    ......................................................................uugcaUaucaccaauguu....................  1   1  AdM
    ......................................................................uugcacaucaccaauguuA...................  68  1  AdM
    ......................................................................uugcCcaucaccaauguuc...................  1   1  AdM
    ......................................................................uugcacauGaccaauguuc...................  2   1  AdM
    ......................................................................Cugcacaucaccaauguuc...................  4   1  AdM
    ......................................................................Augcacaucaccaauguuc...................  1   1  AdM
```

Additional file 11

(c)

```
                                        Star                       Mature
auuaugaggaggauuauaaagaucagguagacuugcuucagaauauugguacugggcaaaaggucacguuuugcacaucaccaauguucugaggcacuucugcugaag
......................................................................uugAacaucaccaauguuc...................   2      1   AdM
......................................................................uugcacaucaccaGuguuc...................   2      1   AdM
......................................................................uugcacaucaccaauguGc...................   6      1   AdM
......................................................................uugcacaucaccaaugCuc...................   6      1   AdM
......................................................................uAgcacaucaccaauguuc...................   1      1   AdM
......................................................................uugcacaucaccaauguuG...................   2      1   AdM
......................................................................uuAcacaucaccaauguuc...................   6      1   AdM
......................................................................uugcacaucaccaauguCc...................   1      1   AdM
......................................................................uugcacaucacAaauguuc...................   6      1   AdM
......................................................................uugcacaucaccaaAguuc...................   1      1   AdM
......................................................................uugcacauAaccaauguuc...................   14     1   AdM
......................................................................uugcacaucaccaCuguuc...................   5      1   AdM
......................................................................uuCcacaucaccaauguuc...................   4      1   AdM
......................................................................uugcacaucaccGauguuc...................   2      1   AdM
......................................................................uugcacaucaccaauAuuc...................   2      1   AdM
......................................................................uugcacaucUccaauguuc...................   1      1   AdM
......................................................................uugcacaucGccaauguuc...................   3      1   AdM
......................................................................uugcacaucaccaauCuuc...................   1      1   AdM
......................................................................uugcacaucaccCauguuc...................   1      1   AdM
......................................................................uugcacaucaccaauguuU...................   70     1   AdM
......................................................................uugcacaucCccaauguuc...................   4      1   AdM
......................................................................uugcGcaucaccaauguuc...................   2      1   AdM
......................................................................uugcacaucaUcaauguuc...................   3      1   AdM
......................................................................uugcaAaucaccaauguuc...................   4      1   AdM
......................................................................uugcacaucaccaauUuuc...................   4      1   AdM
......................................................................uugcacaucaAcaauguuc...................   3      1   AdM
......................................................................uugcacaucaccaaCguuc...................   6      1   AdM
......................................................................uugcacaucaccaaGguuc...................   2      1   AdM
......................................................................uCgcacaucaccaauguuc...................   5      1   AdM
......................................................................uugcacaucaccaauguuc...................   1916   0   AdM
......................................................................uugcacaucaccUauguuc...................   4      1   AdM
......................................................................uugcacaCcaccaauguuc...................   7      1   AdM
......................................................................uugGacaucaccaauguuc...................   1      1   AdM
......................................................................uugcaGaucaccaauguuc...................   2      1   AdM
......................................................................uugcacaGcaccaauguuc...................   6      1   AdM
......................................................................uugcacaucaccaaugAuc...................   4      1   AdM
......................................................................uugcaUaucaccaauguuc...................   5      1   AdM
......................................................................uugcacaucaccaaugGuc...................   13     1   AdM
......................................................................uuUcacaucaccaauguuc...................   27     1   AdM
......................................................................uugcacGucaccaauguuc...................   4      1   AdM
......................................................................uugUacaucaccaauguuc...................   7      1   AdM
......................................................................uGgcacaucaccaauguuc...................   2      1   AdM
......................................................................uugcacaucacUaauguuc...................   3      1   AdM
......................................................................uugcacCucaccaauguuc...................   1      1   AdM
......................................................................uugcacaucaccaaGguucu..................   1      1   AdM
......................................................................uugcacaucaccaauguuUu..................   9      1   AdM
......................................................................uugcacaucaccaaugCucu..................   1      1   AdM
......................................................................uugcacaucaccaaugGucu..................   1      1   AdM
......................................................................uugcacaucaccaaugAucu..................   2      1   AdM
......................................................................uugcacaucaccaauguucC..................   1      1   AdM
......................................................................uuCcacaucaccaauguucu..................   1      1   AdM
......................................................................uugcacaucacUaauguucu..................   1      1   AdM
......................................................................Cugcacaucaccaauguucu..................   1      1   AdM
......................................................................uugcacauAaccaauguucu..................   1      1   AdM
......................................................................uGgcacaucaccaauguucu..................   1      1   AdM
......................................................................uugcacaucaccaauguuAu..................   21     1   AdM
......................................................................uuUcacaucaccaauguucu..................   1      1   AdM
......................................................................uugcacauGaccaauguucu..................   1      1   AdM
......................................................................uugcacaucaccaauguucu..................   142    0   AdM
......................................................................uugcacaucaccaauUuucu..................   3      1   AdM
......................................................................uuAcacaucaccaauguucug.................   2      1   AdM
......................................................................Augcacaucaccaauguucug.................   1      1   AdM
......................................................................uugcacaucaccaauguucuC.................   1      1   AdM
......................................................................uugcacaucaccaauguCcug.................   1      1   AdM
......................................................................uugcacaucaccaGuguucug.................   5      1   AdM
......................................................................uugcacaucaccGauguucug.................   3      1   AdM
......................................................................uugcacaucaccaauguucAg.................   2      1   AdM
......................................................................uugcacaucaccaaugAucug.................   3      1   AdM
......................................................................uugcaAaucaccaauguucug.................   3      1   AdM
......................................................................uugcacaCcaccaauguucug.................   2      1   AdM
```

Additional file 11

(c)

```
                                                       Star                        Mature
auuaugaggaggauuauaaagaucagguagacuugcuucagaauauugguacugggcaaaaggucacguuuugcacaucaccaauguucugaggcacuucugcugaag
......................................................................uugcacaucaUcaauguucug.................   9      1   AdM
......................................................................uugUacaucaccaauguucug.................   8      1   AdM
......................................................................uugcacaucacUaauguucug.................   17     1   AdM
......................................................................uugcacaucaccaaugGucug.................   6      1   AdM
......................................................................uugcacaucaccCauguucug.................   3      1   AdM
......................................................................uugcacaucaccaCuguucug.................   3      1   AdM
......................................................................uGgcacaucaccaauguucug.................   6      1   AdM
......................................................................uuUcacaucaccaauguucug.................   6      1   AdM
......................................................................uugcacaucaccaaCguucug.................   2      1   AdM
......................................................................uugcaGaucaccaauguucug.................   1      1   AdM
......................................................................uugcacaucacGaauguucug.................   1      1   AdM
......................................................................uugcacaucaccaauUuucug.................   5      1   AdM
......................................................................uugcacaucaccaauAuucug.................   9      1   AdM
......................................................................uugcacaucaccaauguGcug.................   4      1   AdM
......................................................................uugcacaucGccaauguucug.................   2      1   AdM
......................................................................uugcacaucaccaauguucug.................   1964   0   AdM
......................................................................uugcacaucaAcaauguucug.................   4      1   AdM
......................................................................uuCcacaucaccaauguucug.................   4      1   AdM
......................................................................uugcacCucaccaauguucug.................   2      1   AdM
......................................................................uugGacaucaccaauguucug.................   1      1   AdM
......................................................................uugcacaucaccUauguucug.................   2      1   AdM
......................................................................Cugcacaucaccaauguucug.................   9      1   AdM
......................................................................uugcacaucCccaauguucug.................   3      1   AdM
......................................................................uugcacaucaccaaGguucug.................   3      1   AdM
......................................................................uugcacUucaccaauguucug.................   2      1   AdM
......................................................................uugcacaucaccaauguucGg.................   5      1   AdM
......................................................................uugcacauAaccaauguucug.................   4      1   AdM
......................................................................uugcacaucaccaauguucuU.................   48     1   AdM
......................................................................uugcacaucaccaauguuUug.................   3      1   AdM
......................................................................uugcacaucaGcaauguucug.................   1      1   AdM
......................................................................uugcacaucaccaaugCucug.................   9      1   AdM
......................................................................uugcCcaucaccaauguucug.................   2      1   AdM
......................................................................uugcacaucaccaauguucuA.................   4      1   AdM
......................................................................uugcacaucaccaUuguucug.................   3      1   AdM
......................................................................uugcGcaucaccaauguucug.................   5      1   AdM
......................................................................uCgcacaucaccaauguucug.................   3      1   AdM
......................................................................uugcacaucacAaauguucug.................   4      1   AdM
......................................................................uugcUcaucaccaauguucug.................   1      1   AdM
......................................................................uugcacGucaccaauguucug.................   2      1   AdM
......................................................................uugcacaucaccaauguuAug.................   1      1   AdM
......................................................................uugcacaucaccaauguucCg.................   1      1   AdM
......................................................................uugcacaGcaccaauguucug.................   3      1   AdM
......................................................................uugcacauGaccaauguucug.................   6      1   AdM
......................................................................uugcaUaucaccaauguucug.................   5      1   AdM
......................................................................uugcacaucaccaCuguucuga................   1      1   AdM
......................................................................uuCcacaucaccaauguucuga................   2      1   AdM
......................................................................uugcacUucaccaauguucuga................   1      1   AdM
......................................................................uugcacaucCccaauguucuga................   3      1   AdM
......................................................................uuAcacaucaccaauguucuga................   1      1   AdM
......................................................................uugcacaucaccaGuguucuga................   2      1   AdM
......................................................................uugcacaucaccaaAguucuga................   2      1   AdM
......................................................................Cugcacaucaccaauguucuga................   3      1   AdM
......................................................................uugcacaucaccaauguucugU................   44     1   AdM
......................................................................uugcacaucaAcaauguucuga................   5      1   AdM
......................................................................uugcacaucaccaaugAucuga................   2      1   AdM
......................................................................uugcacGucaccaauguucuga................   1      1   AdM
......................................................................uugcacaAcaccaauguucuga................   2      1   AdM
......................................................................uugcacaucaccaaugGucuga................   9      1   AdM
......................................................................uugcacaucaccaauguuAuga................   2      1   AdM
......................................................................uugcacaucacUaauguucuga................   9      1   AdM
......................................................................uugcacaucacAaauguucuga................   1      1   AdM
......................................................................uugcacaucaccaauguucGga................   2      1   AdM
......................................................................uugcacaucaccaauguCcuga................   3      1   AdM
......................................................................uugcacaucaccaauguucuga................   1587   0   AdM
......................................................................uugcacaucaccaauguucuAa................   1      1   AdM
......................................................................uugcacauGaccaauguucuga................   7      1   AdM
......................................................................uugcacaucaccaauUuucuga................   1      1   AdM
......................................................................Augcacaucaccaauguucuga................   1      1   AdM
......................................................................uugcaGaucaccaauguucuga................   2      1   AdM
......................................................................uugcacaucaccaauguucuCa................   1      1   AdM
```

Additional file 11

(c)

Star **Mature**

```
auuaugaggaggauuauaaagaucagguagacuugcuucagaauauugguacugggcaaaaggucacguuuugcacaucaccaauguucugaggcacuucugcugaag
......................................................................uugcacaucaccaauguucuUa................   34    1   AdM
......................................................................uugcacaucaccaauguuGuga................   2     1   AdM
......................................................................uugcacaucaccaaGguucuga................   1     1   AdM
......................................................................uugUacaucaccaauguucuga................   7     1   AdM
......................................................................uugcacaucaccaauAuucuga................   6     1   AdM
......................................................................uCgcacaucaccaauguucuga................   3     1   AdM
......................................................................uAgcacaucaccaauguucuga................   2     1   AdM
......................................................................uuUcacaucaccaauguucuga................   9     1   AdM
......................................................................uugcacaucaccaaugCucuga................   5     1   AdM
......................................................................uGgcacaucaccaauguucuga................   3     1   AdM
......................................................................uugcacaucaccGauguucuga................   1     1   AdM
......................................................................uugcacaucaccaauguucCga................   1     1   AdM
......................................................................uugcacaucaccaauguucugC................   3     1   AdM
......................................................................uugcacaucaccaauguucugG................   2     1   AdM
......................................................................uugcacaucGccaauguucuga................   2     1   AdM
......................................................................uugcacaGcaccaauguucuga................   5     1   AdM
......................................................................uugcacaCcaccaauguucuga................   4     1   AdM
......................................................................uugcacaucaUcaauguucuga................   5     1   AdM
......................................................................uugcGcaucaccaauguucuga................   4     1   AdM
......................................................................uugcaAaucaccaauguucuga................   7     1   AdM
......................................................................uugcacaucaccaaCguucuga................   2     1   AdM
......................................................................uugcaUaucaccaauguucuga................   3     1   AdM
......................................................................uugcacaucaccaauguuUuga................   2     1   AdM
......................................................................uugcacauAaccaauguucuga................   5     1   AdM
......................................................................uugAacaucaccaauguucuga................   2     1   AdM
......................................................................uugcacaucaccaauguucugaA...............   26    1   AdM
......................................................................uugcacaucaccaauguucugaC...............   10    1   AdM
......................................................................uugcacaucaccaauguucugaU...............   177   1   AdM
.......................................................................Ggcacaucaccaauguuc...................   1     1   AdM
.......................................................................ugGacaucaccaauguuc...................   1     1   AdM
.......................................................................ugcacaucaccaauguuU...................   2     1   AdM
.......................................................................ugcacaucaccaauguuc...................   18    0   AdM
.......................................................................ugcacaucaccaauguuA...................   1     1   AdM
.......................................................................ugcacaGcaccaauguucu..................   1     1   AdM
.......................................................................ugcacaucaccaauguucu..................   1     0   AdM
.......................................................................ugcacaucGccaauguucug.................   1     1   AdM
.......................................................................ugcaAaucaccaauguucug.................   1     1   AdM
.......................................................................ugcacaucaccaauguucug.................   32    0   AdM
.......................................................................ugUacaucaccaauguucug.................   1     1   AdM
.......................................................................ugcacaucaAcaauguucuga................   1     1   AdM
.......................................................................ugcacaucaccaauguucuUa................   1     1   AdM
.......................................................................ugcacaucaccaauguucuga................   12    0   AdM
.......................................................................ugcacaucaccaauguucugaU...............   2     1   AdM
........................................................................gcacaucaccaauguucug.................   1     0   AdM
........................................................................gcacaucaccaauguucuga................   2     0   AdM
........................................................................gcacaucaccaauguucugaA...............   1     1   AdM
.........................................................................cacaucaccaauguucuga................   1     0   AdM
..........................................................................aAaucaccaauguucuga................   1     1   AdM
..........................................................................acaucaccaauguucugagg..............   1     0   AdM
...........................................................................caucaccaauguucuga................   1     0   AdM
```

Additional file 11

(d)

Provisional ID	: scaffold_1056_34663
Score total	: 2524.8
Score for star read(s)	: 3.9
Score for read counts	: 2518.2
Score for mfe	: 1.2
Score for randfold	: 1.6
Score for cons. seed	:
Total read count	: 4951
Mature read count	: 4816
Loop read count	: 0
Star read count	: 135

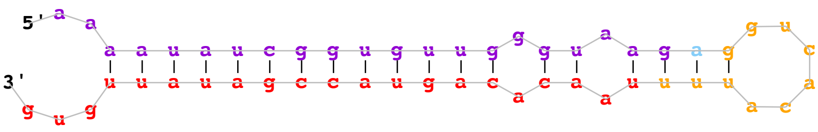

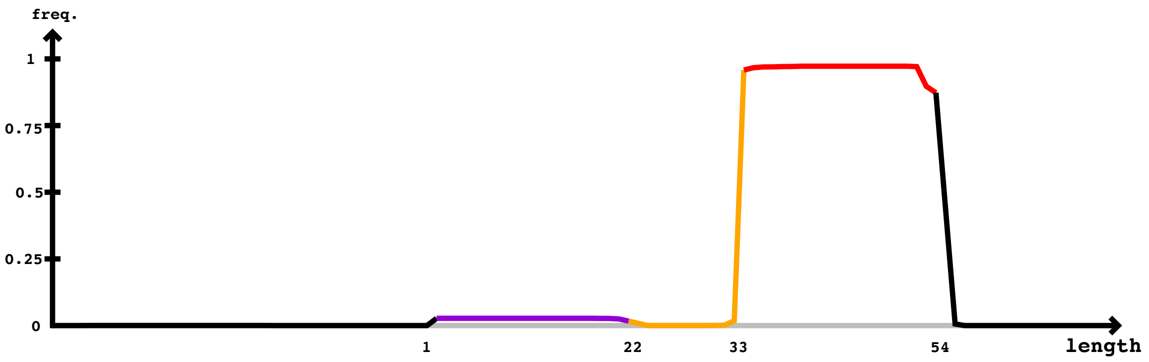


```
                                          Star                               Mature
5'- ggaggcgauguaacucuaccaacaggugcuacugcuucaaaauaucgguguuggguaagaggucacauuuuaacacaguaccgauauugugaagcacguuugcaaauua  -3'   obs
    ggaggcgauguaacucuaccaacaggugcuacugcuucaaaauaucgguguuggguaagaggucacauuuuaacacaguaccgauauugugaagcacguuugcaaauua        exp
    ggaggcgauguaacucuaccaacaggugcuacugcuucaaaauaucgguguuggguaagaggucacauuuuaacacaguaccgauauugugaagcacguuugcaaauua        known
    ((((.........))))(((....)))((.((((((((.(((((((((((((.((.((((......)))).)).))))))))))))).))))))).))..))......    reads   mm   sample
    ..aggcgauguaacucuacca........................................................................................    1       0    AdM
    ......................................aaaauaucgguguuggg......................................................    1       0    AdM
    ......................................aaaauaucggAguuggg......................................................    1       1    AdM
    ......................................aaaauaucgguguugggu.....................................................    1       0    AdM
    ......................................aaaauaucgguguuggCua....................................................    1       1    AdM
    ......................................aaaauaucgguguugggua....................................................    8       0    AdM
    ......................................aaaauaucgguguuggguaa...................................................    39      0    AdM
    ......................................aaaauaucgguguuUgguaa...................................................    1       1    AdM
    ......................................aaaGuaucgguguuggguaa...................................................    1       1    AdM
    ......................................aaaauaucgguguuggguaaC..................................................    3       1    AdM
    ......................................aaaauaucgUuguuggguaag..................................................    1       1    AdM
    ......................................aaaauaucgguguuggguaag..................................................    64      0    AdM
    ......................................aaaauaucgguguuUgguaag..................................................    1       1    AdM
    ......................................aaaaCaucgguguuggguaag..................................................    1       1    AdM
    ......................................aaaauaucgguguuggUuaag..................................................    2       1    AdM
    ......................................aaaauaGcgguguuggguaag..................................................    1       1    AdM
    ......................................aaaauaucggCguuggguaag..................................................    1       1    AdM
    ......................................aaaauaucgguguuggguaaU..................................................    1       1    AdM
    ......................................aaaauaucggugGuggguaag..................................................    1       1    AdM
    ......................................aaaauaucgguguugCguaag..................................................    1       1    AdM
    ......................................aaaauaucgguguuggguaUg..................................................    1       1    AdM
    ......................................aaaauaucggGguuggguaag..................................................    1       1    AdM
    ......................................aaaauaucgguguuggguaaga.................................................    2       0    AdM
    .......................................aaauaucgguguuggguaag..................................................    1       0    AdM
    ...................................................................uAuuaacacaguaccgauauugug..................    1       1    AdM
    ....................................................................uuuaacacaguaccgauauu.....................    1       0    AdM
    ....................................................................uuuaacacaguaccgauauuU....................    1       1    AdM
    ....................................................................uuuaacacaguaccgauauug....................    2       0    AdM
    ....................................................................uuuaacacaguaccgauauugA...................    1       1    AdM
    ....................................................................uuuaacacaguaccgauauugu...................    1       0    AdM
    ....................................................................uuuaacacagAaccgauauugug..................    1       1    AdM
    .....................................................................uuaacacaguaccgauauu.....................    1       0    AdM
```

Additional file 11

(d)

Star Mature

```
ggaggcgauguaacucuaccaacaggugcuacugcuucaaaauaucgguguuggguaagaggucacauuuuaacacaguaccgauauugugaagcacguuugcaaauua
.....................................................................uCaacacaguaccgauauug....................  1     1  AdM
.....................................................................uuaacacaguaccgauCuug....................  1     1  AdM
.....................................................................uuaacacaguaccgauauug....................  5     0  AdM
.....................................................................uuaacacaguaccgauauuU....................  1     1  AdM
.....................................................................uuaacacaguaccgauauugu...................  2     0  AdM
.....................................................................uuaacacaguaccgauauuUug..................  3     1  AdM
.....................................................................uuaacacaguaccgauauCgug..................  1     1  AdM
.....................................................................uuaacacaguaccgauauuguU..................  1     1  AdM
.....................................................................uuaacacaguaccgaGauugug..................  1     1  AdM
.....................................................................uuaacacagGaccgauauugug..................  1     1  AdM
.....................................................................uuaacacaguaccgauauugug..................  44    0  AdM
.....................................................................Cuaacacaguaccgauauuguga.................  2     1  AdM
.....................................................................uuaacacaguaccgauauuguga.................  19    0  AdM
......................................................................uaacacagGaccgauauu.....................  1     1  AdM
......................................................................uaacacaguaccgauauu.....................  5     0  AdM
......................................................................uaacacaguaccgauauug....................  311   0  AdM
......................................................................uaacacaguaccgauauAg....................  1     1  AdM
......................................................................uGacacaguaccgauauug....................  3     1  AdM
......................................................................uaacacaguaccgaGauug....................  2     1  AdM
......................................................................uaacacaguacUgauauug....................  1     1  AdM
......................................................................uaacacagCaccgauauug....................  3     1  AdM
......................................................................uaacacaguaccgauauuA....................  1     1  AdM
......................................................................uaacaUaguaccgauauug....................  2     1  AdM
......................................................................uaGcacaguaccgauauug....................  1     1  AdM
......................................................................uaacacaguaccgauauGg....................  1     1  AdM
......................................................................uaacacaguaUcgauauug....................  4     1  AdM
......................................................................uaacacaguaccgauauuU....................  7     1  AdM
......................................................................uaacacaguaccgauaCug....................  2     1  AdM
......................................................................uaacacaguaccgCuauug....................  2     1  AdM
......................................................................uaacacaguaccgauauuC....................  1     1  AdM
......................................................................uaacacagGaccgauauug....................  3     1  AdM
......................................................................uaaUacaguaccgauauug....................  1     1  AdM
......................................................................uaacGcaguaccgauauug....................  1     1  AdM
......................................................................uaCcacaguaccgauauug....................  1     1  AdM
......................................................................uaacacagAaccgauauug....................  1     1  AdM
......................................................................uaacacaguaccgauauCg....................  1     1  AdM
......................................................................uaacacaguaccgauaGug....................  1     1  AdM
......................................................................uaacacaguaccUauauug....................  1     1  AdM
......................................................................uaacacaguaccCauauugu...................  1     1  AdM
......................................................................uaacacaguaccgauauugu...................  96    0  AdM
......................................................................uaacacaguaUcgauauugu...................  3     1  AdM
......................................................................uaacacaguaccgauaAugu...................  1     1  AdM
......................................................................uaacaUaguaccgauauugu...................  1     1  AdM
......................................................................uaacUcaguaccgauauugu...................  1     1  AdM
......................................................................uaacacaguaccgauauugA...................  5     1  AdM
......................................................................uGacacaguaccgauauugu...................  2     1  AdM
......................................................................uaacacGguaccgauauugu...................  1     1  AdM
......................................................................uaacaAaguaccgauauugu...................  1     1  AdM
......................................................................uaacacaguaccAauauugug..................  1     1  AdM
......................................................................uaaAacaguaccgauauugug..................  7     1  AdM
......................................................................uaacacaguaccgauauAgug..................  1     1  AdM
......................................................................uaacacaguaccgauauGgug..................  4     1  AdM
......................................................................uaacacaguaccgauCuugug..................  1     1  AdM
......................................................................uaaUacaguaccgauauugug..................  11    1  AdM
......................................................................uaacacaguGccgauauugug..................  1     1  AdM
......................................................................uaacaUaguaccgauauugug..................  4     1  AdM
......................................................................uaacacaguaccgUuauugug..................  3     1  AdM
......................................................................uaacacaguaccgauauuguU..................  60    1  AdM
......................................................................uaacacaguaccgauauuUug..................  47    1  AdM
......................................................................uaacacaguaGcgauauugug..................  1     1  AdM
......................................................................uaacacaguaccgCuauugug..................  3     1  AdM
......................................................................uaacaAaguaccgauauugug..................  7     1  AdM
......................................................................uaacacaguaccgauaAugug..................  1     1  AdM
......................................................................uaacacaguacAgauauugug..................  7     1  AdM
......................................................................uaacacaguaccgauauugug..................  2940  0  AdM
......................................................................Aaacacaguaccgauauugug..................  3     1  AdM
......................................................................uaacacaguaccgauauugAg..................  3     1  AdM
......................................................................uaacacagCaccgauauugug..................  26    1  AdM
......................................................................uaacacaguaccgauauuguC..................  6     1  AdM
......................................................................uaacacaguaccgGuauugug..................  3     1  AdM
```

Additional file 11

(d)

```
                                                      Star                          Mature
ggaggcgauguaacucuaccaacaggugcuacugcuucaaaauaucggguguuggguaagaggucacauuuuaacacaguaccgauauugugaagcacguuugcaaauua
.......................................................................uaacacagGaccgauauugug..................   19   1   AdM
.......................................................................uaacacaguaccgauauCgug..................   7    1   AdM
.......................................................................uaacacaguaccgauauuguA..................   1    1   AdM
.......................................................................uGacacaguaccgauauugug..................   27   1   AdM
.......................................................................uaacacaguaccgauauugCg..................   4    1   AdM
.......................................................................uaacacUguaccgauauugug..................   1    1   AdM
.......................................................................Caacacaguaccgauauugug..................   6    1   AdM
.......................................................................uaacacaguaAcgauauugug..................   5    1   AdM
.......................................................................uaacacaguacUgauauugug..................   6    1   AdM
.......................................................................uaacacaguaccgauaCugug..................   4    1   AdM
.......................................................................uaacacaguaccgauauuAug..................   1    1   AdM
.......................................................................uaacacagAaccgauauugug..................   20   1   AdM
.......................................................................uaacacaguacGgauauugug..................   2    1   AdM
.......................................................................uaacacaguaccgaGauugug..................   11   1   AdM
.......................................................................uaacacaAuaccgauauugug..................   3    1   AdM
.......................................................................uaacacCguaccgauauugug..................   1    1   AdM
.......................................................................uaacacaguaccgauGuugug..................   3    1   AdM
.......................................................................uaacacaCuaccgauauugug..................   2    1   AdM
.......................................................................uaacacaguaccUauauugug..................   2    1   AdM
.......................................................................uaacacaUuaccgauauugug..................   3    1   AdM
.......................................................................uaacaGaguaccgauauugug..................   1    1   AdM
.......................................................................uaGcacaguaccgauauugug..................   4    1   AdM
.......................................................................uaacacGguaccgauauugug..................   4    1   AdM
.......................................................................uaacacaguaccgauaGugug..................   5    1   AdM
.......................................................................uaacCcaguaccgauauugug..................   2    1   AdM
.......................................................................uaacUcaguaccgauauugug..................   2    1   AdM
.......................................................................uaacacaguaccgauUuugug..................   2    1   AdM
.......................................................................uaacacaguaccgauauuCug..................   5    1   AdM
.......................................................................uaacacaguaccgauauugGg..................   12   1   AdM
.......................................................................Gaacacaguaccgauauugug..................   1    1   AdM
.......................................................................uaacacaguaccgaAauugug..................   2    1   AdM
.......................................................................uCacacaguaccgauauugug..................   18   1   AdM
.......................................................................uaacGcaguaccgauauugug..................   7    1   AdM
.......................................................................uaacacaguaccgaCauugug..................   3    1   AdM
.......................................................................uaacacaguaUcgauauugug..................   13   1   AdM
.......................................................................uaacacaguaccgauauuUuga.................   2    1   AdM
.......................................................................uaacacagAaccgauauuguga.................   6    1   AdM
.......................................................................uaacacaguaccgauauugAga.................   1    1   AdM
.......................................................................uaacacaguaccgaAauuguga.................   1    1   AdM
.......................................................................uaacacaguaccgauauugugU.................   4    1   AdM
.......................................................................uaacacaguaccgaCauuguga.................   2    1   AdM
.......................................................................uaacGcaguaccgauauuguga.................   3    1   AdM
.......................................................................uaacacaguacAgauauuguga.................   4    1   AdM
.......................................................................uaacacagGaccgauauuguga.................   9    1   AdM
.......................................................................uaacacagCaccgauauuguga.................   2    1   AdM
.......................................................................uaaUacaguaccgauauuguga.................   2    1   AdM
.......................................................................uaacacaguaccgauaCuguga.................   1    1   AdM
.......................................................................uaacacaguaccgauauugugG.................   2    1   AdM
.......................................................................uaacacaguaccgauauuguUa.................   25   1   AdM
.......................................................................uaacacaAuaccgauauuguga.................   1    1   AdM
.......................................................................uaaAacaguaccgauauuguga.................   2    1   AdM
.......................................................................uaacacaguaccgauauCguga.................   3    1   AdM
.......................................................................uaacacaguaccgauauugCga.................   2    1   AdM
.......................................................................uGacacaguaccgauauuguga.................   13   1   AdM
.......................................................................uaacacaguaccgaGauuguga.................   4    1   AdM
.......................................................................uaacacaguaccgauGuguga.................   2    1   AdM
.......................................................................uaacacaguaccgauauuguCa.................   1    1   AdM
.......................................................................uaacacGguaccgauauuguga.................   1    1   AdM
.......................................................................uaacacUguaccgauauuguga.................   1    1   AdM
.......................................................................uaacacaguaccgauauugGga.................   2    1   AdM
.......................................................................uaGcacaguaccgauauuguga.................   3    1   AdM
.......................................................................uaacacCguaccgauauuguga.................   1    1   AdM
.......................................................................uaacacaguacUgauauuguga.................   1    1   AdM
.......................................................................uCacacaguaccgauauuguga.................   8    1   AdM
.......................................................................uaacacaguaccAauauuguga.................   1    1   AdM
.......................................................................uaacacaguaccgauGuuguga.................   1    1   AdM
.......................................................................uaacacaguaUcgauauuguga.................   3    1   AdM
.......................................................................uaacacaguaccgauauuguga.................   688  0   AdM
.......................................................................uaacacaCuaccgauauuguga.................   2    1   AdM
.......................................................................uaacaUaguaccgauauuguga.................   3    1   AdM
```

Additional file 11

(d)

Star **Mature**

```
ggaggcgauguaacucuaccaacaggugcuacugcuucaaaauaucgguguuggguaagaggucacauuuuaacacaguaccgauauugugaagcacguuugcaaauua
......................................................................uaacacaguaAcgauauuguga.................   2    1   AdM
......................................................................uaacaAaguaccgauauuguga.................   2    1   AdM
......................................................................uaacacaguaccgUuauuguga.................   1    1   AdM
......................................................................uaaUacaguaccgauauugugaa................   1    1   AdM
......................................................................uaacacaguacAgauauugugaa................   1    1   AdM
......................................................................uaacacaguaccgauauugugaa................   3    0   AdM
......................................................................uaacacaguaccgauauugugaU................   19   1   AdM
.......................................................................aacacaguaccgauauug....................   2    0   AdM
.......................................................................aacacaguaccgauauugu...................   1    0   AdM
.......................................................................aacacaUuaccgauauugug..................   1    1   AdM
.......................................................................aacacaguacAgauauugug..................   1    1   AdM
.......................................................................aacaUaguaccgauauugug..................   1    1   AdM
.......................................................................aacacaguaccgauauugug..................   29   0   AdM
.......................................................................aacacagCaccgauauuguga.................   1    1   AdM
.......................................................................Cacacaguaccgauauuguga.................   1    1   AdM
.......................................................................aacacaguaccgauauuguga.................   5    0   AdM
.......................................................................aacacaguaccgauauuguUa.................   1    1   AdM
........................................................................acacaguaccgauauug....................   1    0   AdM
........................................................................acacaguaccgauauugug..................   9    0   AdM
........................................................................acacagCaccgauauugug..................   1    1   AdM
........................................................................acacaguaccgauauuguga.................   3    0   AdM
.........................................................................cacaguaccgauauugA...................   1    1   AdM
.........................................................................Uacaguaccgauauugug..................   1    1   AdM
..........................................................................acaguaccgauauugug..................   3    0   AdM
..........................................................................acaguaAcgauauuguga.................   1    1   AdM
..........................................................................acaguaccgauauuguga.................   1    0   AdM
...........................................................................caguaccgauauuguga.................   2    0   AdM
............................................................................aguaccgauauugugaU................   5    1   AdM
```
